# Kurzzeitstrahlentherapie mit 5 × 5 Gy gefolgt von präoperativer Chemotherapie und Resektion bei Patienten mit lokal fortgeschrittenen Rektumkarzinomen führt zu einer Verbesserung des krankheitsfreien Überlebens

**DOI:** 10.1007/s00066-021-01763-8

**Published:** 2021-03-25

**Authors:** Simon Kirste, Emmanouil Fokas, Claus Rödel

**Affiliations:** 1 Klinik für Strahlenheilkunde, Universitätsklinikum Freiburg, Medizinische Fakultät, Albert-Ludwigs-Universität Freiburg, Robert-Koch-Straße 6, 79106 Freiburg, Deutschland; 2 Klinik für Strahlentherapie und Onkologie, Universitätsklinikum Frankfurt, Goethe-Universität Frankfurt, Theodor-Stern-Kai 7, 60590 Frankfurt, Deutschland

## Hintergrund

Die präoperative Radiochemotherapie (RCT), gefolgt von der totalen mesorektalen Exzision (TME), ist eine Standardbehandlung für das lokal fortgeschrittene Rektumkarzinom. In den meisten internationalen Leitlinien wird daran anschließend eine adjuvante Chemotherapie (CT) empfohlen, obwohl deren Verträglichkeit und Durchführbarkeit limitiert sind und ein eindeutiger Vorteil für sie nicht belegt ist. Die Sequenz RCT/TME/(CT) hat zwar zu einer signifikanten Verbesserung der lokalen Kontrolle, nicht jedoch zu einer Verminderung der Fernmetastasierungsrate und damit des Überlebens geführt. Die Kurzzeitstrahlentherapie, gefolgt von einer Kombinationschemotherapie noch vor der Operation (sog. „totale neoadjuvante Therapie“ [TNT]), könnte zu einer besseren Durchführbarkeit der Systemtherapie und somit zu einer Reduzierung der Fernmetastasierung führen.

## Methoden

In die multizentrische, randomisierte Phase-III-Studie (RAPIDO) wurden diejenigen Patienten mit Rektumkarzinom eingeschlossen, bei denen zumindest eines der folgenden MRT-basierten Hochrisikokriterien vorlag: cT4, cN2, Befall der mesorektalen Faszie (MRF+), extramurale Gefäßinvasion (EMVI+), vergrößerte laterale (extramesorektale) Lymphknoten. Die Randomisierung erfolgte in einen Arm mit Kurzzeit-RT (5 × 5 Gy), gefolgt von 6 Zyklen CAPOX oder alternativ 9 Zyklen FOLFOX4 und anschließender TME (experimenteller Arm) oder in einen Arm mit RCT (50,4 Gy in Einzeldosen von 1,8 Gy) mit simultaner Gabe von Capecitabin, gefolgt von TME und einer optionalen adjuvanten Chemotherapie (8 Zyklen CAPOX oder 12 Zyklen FOLFOX4; Standardarm). Der primäre Endpunkt war die Zeit bis zum Auftreten eines krankheitsbezogenen Therapieversagens („disease-related treatment failure“ [DrTF]), definiert als lokoregionäres Rezidiv, Fernmetastase(n), kolorektales Zweitkarzinom oder therapiebedingter Tod. Sekundäre Endpunkte, wie die Therapiedurchführbarkeit, die Akuttoxizität und die Rate an postoperativen Komplikationen, wurden in einer gesonderten Publikation beschrieben.

## Ergebnisse

Von 2011 bis 2016 wurden 920 Patienten eingeschlossen, davon waren 912 auswertbar (462 im experimentellen Arm, 450 im Standardarm). Alle Patienten im experimentellen Arm erhielten die Kurzzeit-RT, bei 84 % konnten zumindest 75 % der konsolidierenden Chemotherapie appliziert werden. Im Standardarm betrug die Compliance für die RCT 93 %, für die postoperative Chemotherapie 58 %.

Die Toxizitäten CTC ≥ Grad 3 im TNT-Arm betrugen insgesamt 48 % und umfassten insbesondere Diarrhö mit 17,6 %; eine Neuropathie trat bei 4,3 % der Patienten auf. Nach Standard-RCT fanden sich bei 25 %, bei adjuvanter Chemotherapie bei 35 % der Patienten Toxizitäten CTC ≥ Grad 3. Die Art der chirurgischen Therapien unterschied sich nicht zwischen den Armen, auch die chirurgischen Komplikationsraten, dokumentiert nach der Clavien-Dindo-Klassifikation, waren nicht signifikant unterschiedlich.

Die pathologische Komplettremissionsrate (pCR) lag mit etwa 28 % im experimentellen Arm versus 14 % nach RCT signifikant höher (*p* < 0,001). Der primäre Endpunkt, DrTF, zeigte im experimentellen Arm eine signifikante Überlegenheit gegenüber der Standard-RCT (nach 3 Jahren 23,7 % versus 30,4 %; HR 0,75; *p* = 0,019); diese Überlegenheit war in allen relevanten Subgruppen, insbesondere auch bei Patienten, die eine postoperative Chemotherapie erhielten, nachweisbar. Die Rate an Fernmetastasen war im experimentellen Arm signifikant niedriger (nach 3 Jahren 20,0 % versus 26,8 %; HR 0,69; *p* = 0,005). Die 3‑Jahres-Lokalrezidivrate nach radikaler Operation einschließlich TME betrug im Standardarm 6,0% und im experimentellen Arm 8,7 % (HR 1,45; *p* = 0,09). Die Lebensqualität war gemäß den EORTC-QLQ-C30-Fragebögen nicht unterschiedlich.

## Schlussfolgerung der Autoren

Die Verbesserung des primären Endpunkts, DrTF, deutet auf eine erhöhte Wirksamkeit der TNT mit 5 × 5 Gy, neoadjuvanter Chemotherapie und TME im Vergleich zur RCT, TME mit oder ohne adjuvante Chemotherapie hin. Daher kann der experimentelle Arm der RAPIDO-Studie als ein neuer Therapiestandard bei lokal fortgeschrittenen Rektumkarzinomen mit MRT-definierten Hochrisikofaktoren gelten.

## Kommentar

Seit der Veröffentlichung der CAO/ARO/AIO-94-Studie ist die präoperative RCT mit simultaner fluorouracilbasierter Chemotherapie, gefolgt von TME und adjuvanter Chemotherapie eine Standardsequenz in der Behandlung des lokal fortgeschrittenen Rektumkarzinoms [1, 2]. Diese Therapie hat zu einer signifikanten Senkung der Lokalrezidivrate geführt, die Rate an Fernmetastasen blieb allerdings unbeeinflusst. Ein möglicher Grund hierfür ist die schlechte Durchführbarkeit der adjuvanten Chemotherapie. Zur Verbesserung des krankheitsfreien Überlebens müsste daher eine Intensivierung und/oder bessere Durchführbarkeit der systemischen Therapiekomponente erreicht werden. Diesem Ziel folgend wurde die sogenannte TNT entwickelt. Dabei wird sowohl die Strahlentherapie als auch die systemische Therapie vor der Resektion appliziert. Ziel dieses Therapieregimes ist insbesondere eine Intensivierung der Chemotherapie zur Senkung von Fernmetastasen bei gleichbleibender lokaler Kontrolle. Als weiterer Effekt der TNT, und des damit einhergehenden verlängerten Intervalls zwischen RT/RCT und Resektion, ist eine erhöhte Rate an Komplettremissionen zu erwarten [3, 4]. Die RAPIDO-Studie hat nun in der Tat diese Hypothesen bestätigt [5, 6].

Ähnlich wie die RAPIDO-Studie hat auch die französische PRODIGE-23-Studie eine Intensivierung der neoadjuvanten Therapie getestet [7]. Sie randomisierte zwischen einer Induktionstherapie mit 6 Zyklen FOLFIRINOX, gefolgt von einer capecitabinbasierten RCT, TME (und weiteren adjuvanten Therapie über drei Monate), und einer capecitabinbasierten RCT, gefolgt von TME und einer adjuvanten FOLFOX- oder Capecitabintherapie über 6 Monate. Die Einschlusskriterien waren weniger präzise definiert als in der RAPIDO-Studie und umfassten Patienten mit T3- („at risk of local recurrence“) oder T4-Tumoren. Im experimentellen Arm war der primäre Endpunkt, das krankheitsfreie Überleben, mit einer HR von 0,69 ebenfalls signifikant verbessert (*p* = 0,03).

Mit der RAPIDO- und der PRODIGE-23-Studie liegen nunmehr also zwei aktuelle randomisierte Phase-III-Studien zum Konzept der intensivierten neoadjuvanten Therapie/TNT vor [5–7]. Beide Studien verwendeten als Kontrollarm eine neoadjuvante fluorouracilbasierte RCT (mit oder ohne adjuvante Chemotherapie), wie sie auch in Deutschland genutzter und leitlinienbasierter bisheriger Standard ist. Beide Studien haben trotz der Verschiedenheit ihrer Designs jeweils den primären Endpunkt erreicht und zeigen eine signifikante und klinisch relevante Verbesserung des krankheitsfreien Überlebens. Übereinstimmend zeigen beide Studien auch, dass sich durch TNT beim fortgeschrittenen Rektumkarzinom eine gegenüber der Standard-RCT mehr als verdoppelte pCR-Rate erreichen lässt.

## Fazit

Auf Grundlage dieser Studien legt daher ein konsentiertes Statement der ACO, AIO und ARO nahe, dass die TNT als präferierte neue Therapieoption bei Patienten mit einem lokal weit fortgeschrittenen Rektumkarzinom gelten kann, wobei die RT entweder als RCT oder als Kurzzeit-RT erfolgen kann [8]. Zur Frage der optimalen Sequenz der TNT lieferten die amerikanische OPRA-Studie [9] und die CAO/ARO/AIO-12 [3] Hinweise, dass die Sequenz RCT/CT der umgekehrten Reihenfolge insbesondere hinsichtlich der lokalen Wirksamkeit überlegen ist.

Die kürzlich gestartete ACO/ARO/AIO-18.1-Studie (ClinicalTrials.gov Identifier: NCT04246684) soll nun klären, ob die TNT mit 5 × 5 Gy gefolgt von CAPOX/FOLFOX gemäß der RAPIDO-Studie oder die TNT mit optimierter RCT gefolgt von CAPOX/FOLFOX analog OPRA bzw. CAO/ARO/AIO-12 den primären Endpunkt Organerhalt ohne TME günstiger beeinflusst.

*Simon Kirste, Emmanouil Fokas und Claus Rödel,*

*Freiburg und Frankfurt/M.*
